# Evaluation of standard and modified two-tiered testing algorithms using well-characterized early Lyme disease samples

**DOI:** 10.1128/jcm.01187-25

**Published:** 2026-04-21

**Authors:** Elizabeth J. Horn, Barry Menefee, Anna M. Schotthoefer, George Dempsey, Matt McArdle, Allison F. Weber, Cathy De Luca, Bobbi S. Pritt, John A. Branda

**Affiliations:** 1 Lyme Disease Biobank, Portland, Oregon, USA; 2 Gold Standard Diagnostics, Davis, California, USA; 3 Marshfield Clinic Research Institute 513992 , Marshfield, Wisconsin, USA; 4 East Hampton Family Medicine, East Hampton, New York, USA; 5 Stony Brook University 12301, Stony Brook, New York, USA; 6 Mayo Clinic 6915, Rochester, Minnesota, USA; 7 Massachusetts General Hospital 2348, Boston, Massachusetts, USA; Vanderbilt University Medical Center, Nashville, Tennessee, USA

**Keywords:** Lyme disease, serology, two-tiered testing, diagnostic algorithms, STTT, MTTT, seroconversion, biorepository, biobank

## Abstract

Current laboratory testing for Lyme disease (LD) relies on serology. We evaluated the performance of standard two-tiered testing (STTT) and modified two-tiered testing (MTTT) algorithms using samples obtained from well-characterized patients with early LD in the U.S. East Coast and Upper Midwest. Participants with signs and symptoms of early LD (cases) and controls were enrolled by Lyme Disease Biobank. We compared the performance of four FDA-cleared STTT or MTTT algorithms using serum samples from 251 participants (107 cases, 69 with a convalescent draw; 144 endemic controls). At the initial blood draw, algorithm sensitivity ranged from 22% to 36%, with specificity ranging from 98% to 100%. MTTT algorithms showed higher sensitivity compared with STTT algorithms (*P* ≤ 0.05). One STTT algorithm was less sensitive than the other (*P* = 0.035), and there was no significant difference in sensitivity between MTTT algorithms. There was also discordance between algorithms; only 22 of the 45 samples classified as laboratory confirmed by Lyme Disease Biobank testing were positive using all algorithms evaluated. Likelihood of positive two-tiered serology among cases with a suspected erythema migrans (EM) skin lesion increased with longer lesion duration and/or when presenting with >1 constitutional symptom. Participants with suspected EM without constitutional symptoms were unlikely to test positive by any algorithm evaluated. Testing convalescent samples did not improve LD detection, and seroconversion was rare. While MTTT confirmed more early LD cases than STTT, all two-tiered algorithms evaluated were insensitive in this population. Novel diagnostics that improve laboratory confirmation for early LD are urgently needed.

**IMPORTANCE:**

This study confirms that two-tiered serologic testing algorithms are insensitive in early Lyme disease, particularly within the first 2 weeks after symptom onset or when erythema migrans is not accompanied by constitutional symptoms. It also demonstrates that seroconversion is rare after antibiotic treatment. These results highlight the need for novel diagnostics for early Lyme disease that do not rely on serologic testing.

## INTRODUCTION

Lyme disease (LD) is caused by bacteria in the *Borrelia burgdorferi sensu lato* complex and transmitted through the bite of an infected *Ixodes* (black-legged) tick. LD is the most common vector-borne disease in the U.S. and a significant public health threat (1, 2). Early LD, with signs and symptoms of short duration (up to 30 days) at the time of initial clinical recognition, may present with non-specific constitutional symptoms resembling viral illness, including headache, fatigue, arthralgia, neuralgia, and fever, but without respiratory symptoms (1, 3). Approximately 70% of patients present with erythema migrans (EM), an annular, expanding, erythematous skin lesion (1, 3). While concentric rings (“bulls-eye rash”), or at least central clearing, are often emphasized and are readily recognized, it is not the most common presentation of EM (4, 5). Prompt diagnosis and treatment are key to avoiding later, more severe manifestations or complications of LD (6–8). For patients presenting with suspected EM in areas of endemicity, treatment is initiated based on a clinical diagnosis, and laboratory testing is not recommended because the results are likely to be falsely negative (7, 9, 10). For patients presenting with symptoms compatible with early LD and without EM, an accurate clinical diagnosis relies on positive laboratory testing, but negative test results do not rule it out.

Current laboratory testing for LD uses serology, an indirect method that detects the host response to infection. However, an antibody response to *B. burgdorferi* is often not detectable during a “window period” of several days to a few weeks after infection (10). Early in the humoral immune response, IgM-class antibodies frequently develop against outer surface protein C (OspC), variable major protein-like sequence, expressed (VlsE), and flagellar protein B, and sometimes other antigens, including flagellar protein A, decorin binding proteins A and B, RevA, p66, BBK07, BBK32, BBG33, LA7, BmpA, FliL, and several oligopeptide permeases (OppA1, OppA2, and OppA4) (10). IgG antibodies are reliably detectable approximately 1 to 2 months after infection without antibiotic treatment. Commercially available serologic assays target combinations of these antigens expressed early in infection, particularly VlsE and OspC.

The standard two-tiered testing (STTT) algorithm relies on a first-tier enzyme immunoassay (EIA). When the first-tier test is positive or equivocal, second-tier confirmatory IgM and IgG immunoblotting is performed and interpreted using specific criteria. A positive IgM immunoblot requires at least 2 of these 3 bands, 23, 39, and 41 kDa, to be present; IgG positivity requires a minimum of 5 of these 10 bands, 18, 23, 28, 30, 39, 41, 45, 58, 66, and 93 kDa (9–11). To improve diagnostic specificity, it is recommended that IgM immunoblot positivity only be considered diagnostic within the first 30 days of symptoms. After 30 days, only a positive IgG immunoblot should be considered diagnostic. In 2019, the CDC approved a modified two-tiered testing (MTTT) algorithm as an acceptable alternative to STTT. While MTTT also uses a first-tier EIA, the second tier consists of one or two EIAS that recognize antigens distinct from those used on the first tier (12). MTTT algorithms have been shown to have higher sensitivity than STTT algorithms in early stages of infection (13). MTTT does not rigidly subscribe to the “one-month” rule; IgM positivity can be considered diagnostic after 30 days of symptoms, although clinical correlation is still advised (10).

Lyme Disease Biobank (LDB) provides samples collected from well-characterized LD cases and controls to investigators developing more accurate diagnostics for LD and other tick-borne infections (TBI). As part of LDB’s characterization, testing was performed on acute- and (if obtained) convalescent-phase serum samples using a first-tier IgM/IgG ELISA from Zeus Scientific and second-tier IgM and IgG immunoblots from Viramed. In the current study, early LD and control samples from LDB were analyzed with additional commercially available serologic assays from Gold Standard Diagnostics and Zeus Scientific. Results of individual serologic assays were retrospectively used to model the performance (sensitivity and specificity) of STTT and MTTT algorithms. The overall goal was to assess the clinical utility of serologic testing and to compare the performance of MTTT and STTT using a real-world, prospectively archived, early LD sample collection.

## MATERIALS AND METHODS

### Inclusion criteria for enrollment in LDB

LDB enrolled participants based on signs and symptoms of early LD, with or without clinically suspected EM, and also enrolled control participants living in regions of endemicity (14, 15). Inclusion and exclusion criteria are summarized in Table 1. LD cases were enrolled at a single site in East Hampton (EH), New York, from 2018 to 2020 and several sites in Wisconsin (WI) from 2017 to 2019, including Lake Hallie, Marshfield, Minocqua, Wausau, and Weston. Endemic controls without a history of LD or other TBI were enrolled from the same sites during the same collection seasons. Participants with suspected early LD were given an optional opportunity to provide a convalescent (second) blood sample 2–3 months after the initial draw. Clinical data, including demographics, information about signs and symptoms of early LD, and whether antibiotics were prescribed, were collected using case report forms. Participants from EH were enrolled under Advarra IRB protocol Pro00012408, and participants from WI were enrolled under Marshfield Clinic Research Institute IRB protocol SCH20216. In total, 179 cases (109 with convalescent draws) and 148 endemic controls were enrolled at EH from 2018 to 2020 and at WI from 2017 to 2019.

**TABLE 1:** Lyme Disease Biobank study participant inclusion and exclusion criteria

Enrollment type	Inclusion criteria	Exclusion criteria
Enrolled with suspected EM	Physician identification of EM/annular expanding lesion	Immunocompromised <10 yr of age Antibiotics initiated >48 h Tick bite reaction only
Enrolled without EM	Physician suspicion of LD At least one of the following: headache, fatigue, fever, chills, joint pain, or muscular pain Suspected tick exposure/tick bite	Immunocompromised <10 yr of age Antibiotics initiated >48 h History of chronic fatigue syndrome, rheumatologic disease, multiple sclerosis
Endemic controls	Generally healthy individuals	Immunocompromised <10 yr of age History of LD or TBI

### LDB sample characterization

As part of LDB’s characterization, blinded serologic testing was performed on samples from cases (first and second draws) and controls (single draw) using FDA-cleared serologic assays at the end of each collection season. Serologic testing was performed using (i) a VlsE/pepC10 IgM/IgG ELISA (Zeus Scientific, Raritan, NJ), followed by IgM and IgG ViraStripe immunoblots (Viramed; Biotech AG, Germany) at Mayo Clinic (MC, Rochester, MN); and (ii) a C6 peptide ELISA (Oxford Immunotec, Marlborough, MA) performed at Stony Brook University (SB, Stony Brook, NY). STTT algorithms were retrospectively applied using VlsE/PepC10 ELISA and IgM and IgG immunoblots, all from MC, and C6 peptide ELISA from SB and IgM and IgG immunoblots from MC. Real-time PCR of whole blood for *Borrelia* (*Borrelia burgdorferi, Borrelia mayonii,* and *Borrelia miyamotoi*) was also performed on cases and controls (first draws only) at MC. These test results were then used to classify the cases as having Laboratory Confirmed (LC) Lyme disease, Probable Lyme disease, Suspected Lyme disease, or Symptomatic no Lesion (14, 15). These classifications are used when selecting samples to distribute to investigators and to help distinguish between samples from participants with clinical versus clinical and laboratory evidence of LD.

### Inclusion criteria for current study

For this study, 107 cases (69 with a second draw) and 144 endemic controls (single draw) were included. Inclusion as a case required either classification as LC by LDB laboratory testing (*n* = 45; further described below), a suspected EM lesion >5 cm with negative confirmatory laboratory testing results (*n* = 58), or a positive first-tier VlsE/pepC10 serology result in cases enrolled with suspected EM ≤5 cm or without EM (*n* = 4). Possible cases (*n* = 72) enrolled with suspected EM ≤5 cm or without EM that were negative on the first-tier VlsE/pepC10 ELISA were excluded. Controls (*n* = 4) with insufficient serum inventory were also excluded.

The 45 LDB LC Lyme cases were classified using the following criteria: 32 participants with suspected early LD who had STTT-positive first-draw samples with VlsE/pepC10 (*n* = 31) and/or C6 peptide ELISAs (*n* = 28) as the first-tier test; 10 participants with EM >5 cm whose first-draw samples were positive using both VlsE/pepC10 and C6 peptide ELISAs but negative using IgM and IgG immunoblots; 2 participants whose convalescent phase samples were IgG immunoblot positive when the first draw results had been negative regardless of convalescent-phase first-tier results (IgG seroconversion); and 1 participant whose first-draw whole blood sample was PCR positive for *B. burgdorferi* (14, 15).

### EM status and demographics for current study

Of the 107 cases, 93 presented with a suspected EM (91 of 93 had skin lesions measuring >5 cm), and 14 presented with constitutional symptoms compatible with early LD but without EM. Sixty-four percent (69/107) of cases provided an optional convalescent draw 2–3 months after the initial draw. The mean age of cases was approximately 10 years older than the controls; there was a higher percentage of male cases than controls (65% vs 24%); and the majority of cases and controls were white (85% vs 88%) (Table 2). Demographics were consistent with the overall LDB early LD cohort, including the larger proportion of males enrolled as cases (14, 15).

**TABLE 2:** Demographic information collected at enrollment from cases and controls^*a*^^,^^*b*^

	All cases	Endemic controls
	*N* = 107	*N* = 144
	N (%)	N (%)
Site		
East Hampton (EH)	54 (50)	34 (24)
Wisconsin (WI)	53 (50)	110 (76)
Sex		
Male	70 (65)	35 (24)
Female	37 (35)	109 (76)
Race		
Asian	0 (0)	7 (4)
Hispanic or Latino	16 (15)	11 (8)
White	91 (85)	126 (88)
Age		
Mean (median)	55 (56)	43 (42)
Range	11–88	12–90

^*a*^ Of the 107 cases, 93 (87%) were enrolled with suspected EM, with 91 of 93 (98%) enrolled with suspected EM >5 cm.

^*b*^ EM, erythema migrans; EH, East Hampton, NY; WI, Marshfield, WI, which includes samples from Lake Hallie, Marshfield, Minocqua, Wausau, and Weston. Samples included in this study were collected in EH (2018–2020) and WI (2017–2019).

### Algorithmic testing and comparisons for current study

For this study, all 320 serum samples (107 case and 144 control first draws; 69 case second draws) were tested blindly at Gold Standard Diagnostics (GSD, Davis, CA) using the following FDA-cleared assays: first-tier IgG/IgM VlsE-OspC ELISA, followed by second-tier IgM, IgG, and IgG+IgM ELISAs, and IgM and IgG immunoblots, with kits manufactured by GSD (Frankfurt, Germany). LDB LC Lyme cases (*n* = 45) and endemic controls (*n* = 144) were also tested blindly with FDA-cleared second-tier IgM and IgG ELISAs (Zeus Scientific). Using these test results, STTT and MTTT results were determined by retrospectively applying the two-step algorithms according to CDC guidance (11, 12). GSD MTTT interpretation was based on first-tier IgG/IgM VlsE-OspC ELISA results and second-tier GSD IgM and IgG ELISA results produced by employees of GSD. Zeus MTTT interpretation was based on first-tier VlsE/pepC10 IgM/IgG ELISA results produced by MC and second-tier Zeus ELISA results produced by employees of GSD.

Performance was evaluated by calculating the sensitivity and specificity of the two-tiered testing algorithms based on STTT or MTTT interpretative criteria for all samples. Agreement between testing algorithms was evaluated by calculating the percentages of positive and negative test agreement and the kappa statistic based on 2 × 2 contingency tables for each test comparison. The significance of the differences between proportions in the tables was determined based on McNemar’s test. The following two-tiered testing algorithms were evaluated:

- MC STTT: first-tier VlsE/pepC10 IgM/IgG ELISA manufactured by Zeus Scientific, followed by ViraStripe IgM and IgG immunoblots manufactured by Viramed.
- GSD STTT: first-tier IgG/IgM VlsE-OspC ELISA, followed by IgM and IgG immunoblots (all assays manufactured by Gold Standard Diagnostics).
- GSD MTTT: first-tier IgG/IgM VlsE-OspC ELISA, followed by second-tier IgM and IgG ELISAs; IgG+IgM second-tier ELISA was not used to calculate the two-tiered testing algorithm (all assays manufactured by Gold Standard Diagnostics).
- Zeus MTTT: first-tier VlsE/pepC10 IgM/IgG ELISA, followed by second-tier IgM and IgG ELISAs (all assays manufactured by Zeus Scientific).

Two-tiered algorithm performance was also stratified based on the presence or absence of constitutional symptoms at the time of initial blood draw. Participants enrolled as cases were specifically asked about these nine symptoms: body aches, chills, fatigue, fever, headache, joint pain, nausea, neuralgia, and night sweats. Performance among the subgroup of participants with constitutional symptoms with and without EM was compared with performance among the subgroup of participants with EM but no constitutional symptoms using odds ratios (OR), Fisher’s exact, and Mann-Whitney U tests. Performance among the subgroup of participants enrolled with EM was compared with performance among the subgroup of participants without EM using chi-squared and Mann-Whitney U tests. Statistical analyses were conducted using SAS software, version 9.4 (SAS Institute, Inc., Cary, NC), and R (R version 4.4.0) using the tidyverse package for data manipulation and ggplot for figures.

## RESULTS

### First draw results and two-tiered testing algorithm comparisons

For all 107 early LDB cases evaluated in this study, the sensitivity of the two-tiered testing algorithms was low, ranging from 22% for GSD STTT, 29% for MC STTT, and 36% for GSD MTTT (Table 3). The Zeus MTTT algorithm is not included in these comparisons because data were only available for the subset of LDB LC cases. The GSD MTTT algorithm was more sensitive than either STTT algorithms (*P* < 0.05), with GSD MTTT vs MC STTT (*P* = 0.052) and GSD MTTT vs GSD STTT (*P* < 0.001). When comparing STTT algorithms, GSD STTT was less sensitive than MC STTT (*P* = 0.035). Individual assay results are available in Table S1.

**TABLE 3:** Performance of STTT, MTTT, and first-tier assays at initial draw^*a*^^,^^*b*^

	Sensitivity			Specificity
	All cases	Cases enrolled w/EM	LDB LC Lyme cases	Endemic controls
	*N* = 107	*N* = 93	*N* = 45	*N* = 144
	TP, % (95% CI)	TP, % (95% CI)	TP, % (95% CI)	TN, % (95% CI)
Two-tiered algorithms				
MC STTT	31, 29% (20%–38%)	22, 24% (15%–33%)	31, 69% (55%–82%)	144, **100%** (100%–100%)
GSD STTT	24, 22% (14%–30%)	19, 20% (12%–29%)	24, 53% (39%–68%)	144, **100**% (100%–100%)
GSD MTTT	38, **36**% (26%–45%)	30, **32**% (23%–42%)	37, **82**% (71%–93%)	144, **100%** (100%–100%)
Zeus MTTT	NA	NA	37, **82**% (71%–93%)	141, 98% (96%–100%)
First-tier assays				
VlsE/pepC10 ELISA (Zeus)	54, **50**% (41%–60%)	41, **44**% (34%–54%)	42, **93**% (86%–100%)	127, 88% (83%–93%)
VlsE-OspC ELISA (GSD)	40, 37% (28%–47%)	31, 33% (24%–43%)	37, 82% (71%–93%)	144, **100%** (100%–100%)

^*a*^ For sensitivity, number testing positive, % (95% CI), and for specificity, number testing negative, % (95% CI), are shown. In addition to all cases, two subsets of cases (enrolled with suspected EM or LC Lyme cases) are shown. The tests with the highest sensitivity or specificity for each subset of participants are indicated in bold in each column. For the two-tiered algorithms, VlsE/pepC10 ELISA is the first-tier for MC STTT and Zeus MTTT, while VlsE-OspC ELISA is the first-tier for GSD STTT and GSD MTTT.

^*b*^ MC, Mayo Clinic; GSD, Gold Standard Diagnostics; Zeus, Zeus Scientific; STTT, standard two-tiered testing; MTTT, modified two-tiered testing; TP, true positive; TN, true negative; NA, not applicable; LDB LC, Lyme Disease Biobank lab confirmed; EM, erythema migrans.

Among the 45 LDB LC Lyme cases, more were positive by MTTT than STTT, with 33 (73%) testing positive by one or both STTT algorithms compared to 40 (89%) testing positive by one or both MTTT algorithms (*P* = 0.020). Zeus MTTT was significantly more sensitive than MC STTT (*P* = 0.034) or GSD STTT (*P* = 0.001). GSD MTTT was also significantly more sensitive than GSD STTT (*P* < 0.001) but not MC STTT (*P* = 0.083) in this subset of cases. When comparing MTTT algorithms, there was no significant difference in sensitivity between GSD MTTT and Zeus MTTT algorithms (*P* = 1).

For the 33 LDB LC Lyme cases that were positive by STTT, 31 were positive with MC, 24 were positive with GSD, and 22 were positive with both STTT algorithms. For the 40 LDB LC cases that were positive by MTTT, 37 were positive with GSD tests, 37 were positive with Zeus tests, and 34 were positive with both manufacturers’ MTTT algorithms. Only 22/45 (49%) of the LDB LC Lyme samples tested positive with all four two-tiered testing algorithms evaluated. Four LDB LC Lyme cases did not test positive by any STTT or MTTT using the first draw blood sample, including two with EM >5 cm that were positive using both VlsE/pepC10 and C6 ELISAs, one that seroconverted, and one sample that was blood PCR positive. Results for each of the 45 LDB LC Lyme samples are shown in Table S2. Among the 62 suspected LD cases not designated as lab confirmed by LDB, only one sample tested positive by GSD MTTT (Zeus second-tier assays were not run on this sample), and none were positive by either STTT algorithm.

The two-tiered algorithms evaluated had high specificity (98%–100%) using LDB endemic control samples. While MC STTT, GSD STTT, and GSD MTTT did not identify any positives among the endemic controls, 3 of 144 control samples (2%) were positive by Zeus MTTT, although this difference in specificity was not statistically significant (Table 3).

There was substantial to excellent overall agreement among the two-tiered algorithms: the kappa statistic value was 0.74 (95% CI: 0.62–0.87) between GSD STTT and GSD MTTT, 0.76 (95% CI: 0.63–0.89) between GSD STTT and MC STTT, 0.80 (95% CI: 0.69–0.91) between GSD MTTT and MC STTT, and 0.85 (95% CI: 0.76–0.95) between GSD MTTT and Zeus MTTT. There was also high negative test agreement across the two-tiered testing algorithms. Of the 85 LD cases that were negative on at least one two-tiered algorithm, 65 (76%) were negative on all algorithms, and 141 of 144 controls (98%) were negative on all two-tiered algorithms (Zeus MTTT was positive on three controls).

Among the two different manufacturers’ first-tier assays, Zeus VlsE/pepC10 ELISA demonstrated greater sensitivity (*P* = 0.001), and GSD VlsE-OspC ELISA demonstrated greater specificity (*P* < 0.001) (Table 3). Of the 107 LD cases evaluated, only 38 cases (36%) were positive by both of these first-tier ELISAs at the initial draw, compared to 54 (50%) positive with VlsE/pepC10 ELISA and 40 (37%) positive with VlsE-OspC ELISA individually. Additionally, 44 LD cases were positive by C6 peptide ELISA, and 35 of these cases were also positive with VlsE/pepC10 ELISA and VlsE-OspC ELISA.

### Convalescent draw results

Sixty-nine participants with LD, including 59 (86%) enrolled with suspected EM >5 cm, returned for a convalescent draw 2–3 months after the initial draw. Of the 69 samples collected at the convalescent draw, 7% were positive by MC STTT, 4% by GSD STTT, and 22% by GSD MTTT (Table 4). These samples were not evaluated with the Zeus MTTT algorithm, and only IgG blots were considered in the STTT algorithms, as the convalescent draws were collected beyond 30 days of illness. None of the 69 participants seroconverted using either of the STTT algorithms. Of the 15 LD cases that were GSD MTTT positive at the convalescent draw, 13 (87%) were positive by GSD MTTT at the initial draw. The two additional cases identified by GSD MTTT seroconversion were enrolled with suspected EM >5 cm and were positive by Zeus VlsE/pepC10 ELISA, but were not positive by any two-tiered algorithm at the initial draw. Seroconversion, by any individual immunoblot or ELISA test, was rare—ranging from 1% to 4% depending on the assay (Table 4). Individual assay results are available in Table S1.

**TABLE 4:** Seroconversion results at the convalescent (second) draw^*a*^^,^^*b*^

	Cases positive at initial draw	Cases positive at second draw		Seroconversion
	*N* = 69	*N* = 69		*N* = 69
	N (%)	N (%)	N (%)	N (%)
Two-tiered algorithms		IgM or IgG	IgG-only	
MC STTT	20 (29%)	19 (28%)	5 (7%)	0 (0%)
GSD STTT	15 (22%)	14 (20%)	3 (4%)	0 (0%)
GSD MTTT	22 (32%)	15 (22%)	NA	2 (4%)
Second-tier assays				
MC Blot—IgM	22 (32%)	20 (29%)		4 (6%)
GSD Blot—IgM	14 (20%)	13 (19%)		5 (7%)
GSD ELISA—IgM	20 (29%)	16 (23%)		2 (3%)
MC Blot—IgG	6 (9%)	7 (10%)		2 (3%)
GSD Blot—IgG	5 (7%)	4 (6%)		1 (1%)
GSD ELISA—IgG	13 (19%)	10 (14%)		2 (3%)
GSD ELISA—IgG+IgM	22 (32%)	20 (29%)		3 (4%)

^*a*^ For STTT algorithms and MC and GSD immunoblots, seroconversion is defined as the change from a negative result at the initial draw to a positive result at the convalescent draw. For the GSD MTTT algorithm and ELISAs, seroconversion is defined as the change from a negative result at the initial draw to a positive or equivocal result at the convalescent draw. Convalescent draws were taken 2–3 months after the initial draw. Zeus second-tier assays were not performed on convalescent samples.

^*b*^ MC, Mayo Clinic; GSD, Gold Standard Diagnostics; STTT, standard two-tiered testing; MTTT, modified two-tiered testing; ELISA, enzyme-linked immunosorbent assay; NA, not applicable.

### Clinical features and positive results using two-tier testing algorithms

At the time of enrollment, participants enrolled as LD cases were asked if they were currently experiencing any of nine named symptoms associated with early LD. Sixty-seven percent (72/107) reported experiencing at least one of these symptoms (mean 3.6 symptoms; median 4 symptoms). The four most common symptoms among the cases were fatigue (*n* = 57), body aches (*n* = 56), headache (*n* = 49), and joint pain (*n* = 49). Presenting with at least one symptom at enrollment was significantly associated with testing positive by any of the two-tiered algorithms evaluated in this study (*P* < 0.001). Cases testing positive using any of these algorithms reported more symptoms (mean 5.3 vs 2.4) and for a longer duration (mean 8.3 vs 3.3 days) compared to those testing negative using all four algorithms. For each two-tiered testing algorithm, participants testing positive had more symptoms and had symptoms for longer (Table 5). Likewise, the odds of testing positive using each algorithm were much higher when enrolled with constitutional symptoms with or without EM (Table 6).

**TABLE 5:** Symptom comparison between two-tiered algorithms^*a*^^,^^*b*^

Two-tiered algorithms	Tested positive			Tested negative			Significant difference?	
	N	Number of symptoms	Days with symptoms	N	Number of symptoms	Days with symptoms	Number of symptoms	Days with symptoms
		Mean (median)	Mean (median)		Mean (median)	Mean (median)	*P* value	*P* value
MC STTT	31	5.8 (6)	8.7 (6)	76	2.6 (1)	3.8 (2)	***	***
GSD STTT	24	5.5 (5.5)	10.0 (7)	83	2.0 (3)	3.9 (2)	***	***
GSD MTTT	38	5.2 (5)	8.8 (6.5)	69	2.7 (1)	3.3 (1)	***	***
Zeus MTTT	37	5.6 (6)	8.8 (6.5)	8	4.8 (5)	3.4 (2.5)		*

^*a*^ Of the 72 cases enrolled with symptoms, 68 (94%) reported the number of days they had been experiencing symptoms (0 to 30 days). For each two-tiered algorithm, two Mann-Whitney U tests indicated if there were significant differences in the number of days with symptoms between those who tested positive and those who tested negative. The stars indicate the level of significance: *P < 0.05; **P < 0.01; ***P < 0.001.

^*b*^ MC, Mayo Clinic; GSD, Gold Standard Diagnostics; Zeus, Zeus Scientific; STTT, standard two-tiered testing; MTTT, modified two-tiered testing.

**TABLE 6:** Odds of testing two-tiered positive with symptoms^*a*^^,^^*b*^

Two-tiered algorithms	Odds of testing positive when enrolled with symptoms	
	OR	95% CI
MC STTT	24.3	3.2–187.3
GSD STTT	16.0	2.1–123.9
GSD MTTT	10.1	2.8–36.0
Zeus MTTT	5.83	0.68–49.72

^*a*^ The ORs indicate the odds of testing positive by two-tiered algorithms at enrollment when presenting with constitutional symptoms compared to the odds of testing positive without constitutional symptoms. All the ORs except for Zeus MTTT are significant, *P* < 0.001 by Fisher’s exact test.

^*b*^ MC, Mayo Clinic; GSD, Gold Standard Diagnostics; STTT, standard two-tiered testing; MTTT, modified two-tiered testing; OR, odds ratio.

**Fig 1:**
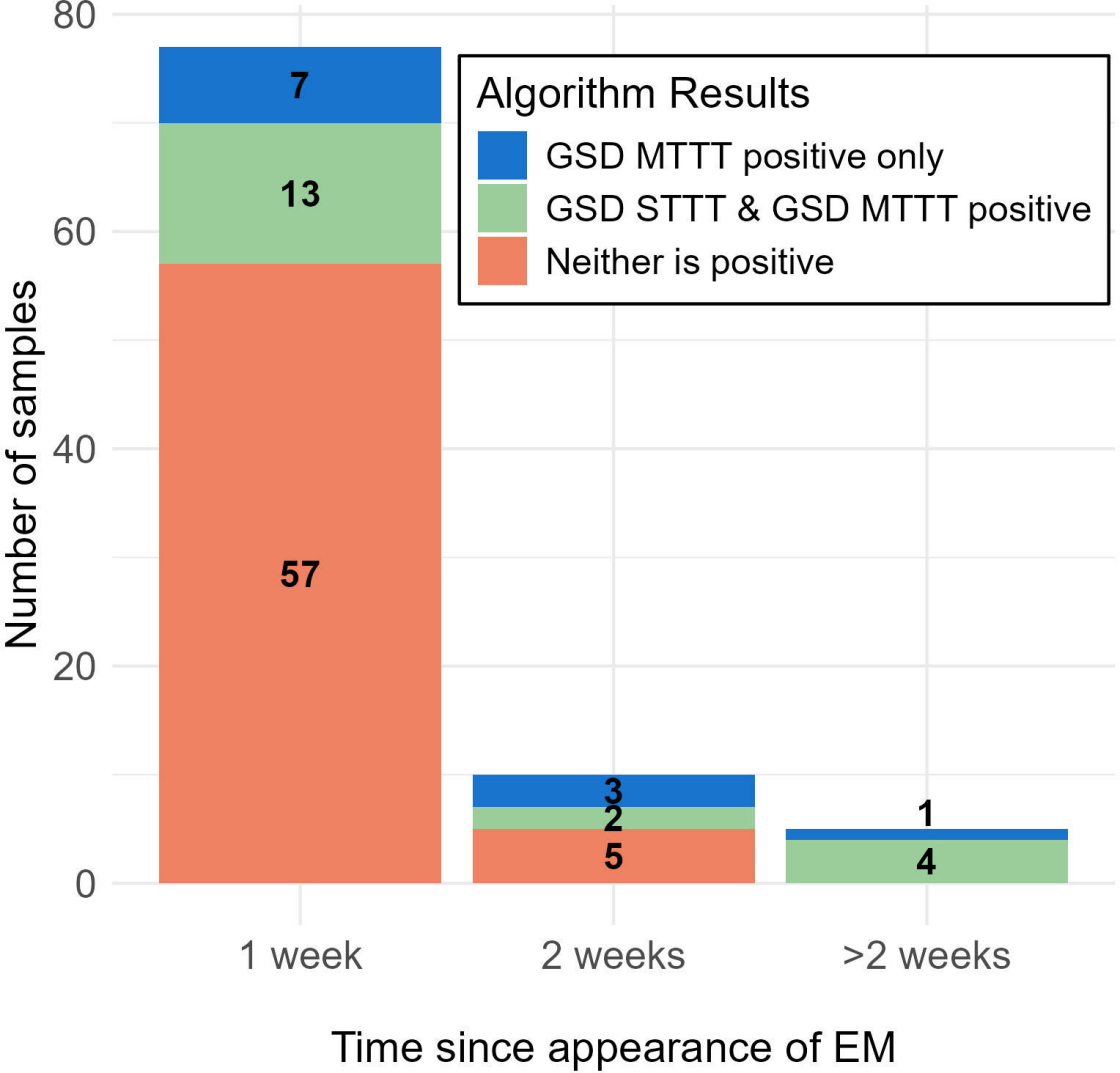
Positive GSD MTTT and STTT algorithm results in samples collected from participants enrolled with suspected EM. MTTT, modified two-tiered testing; STTT, standard two-tiered testing; EM, erythema migrans.

Similarly, LD cases enrolled with suspected EM (*n* = 93) were more likely to test positive by STTT or MTTT if they also had constitutional symptoms. Very few of the participants enrolled with suspected EM who were otherwise asymptomatic (*n* = 35) tested positive by STTT (*n* = 1) or MTTT (*n* = 3). For the 92 cases enrolled with suspected EM with information about EM duration, the likelihood of a positive two-tiered testing result increased according to the duration that the suspected EM lesion had been present prior to the initial blood draw. Those positive by any two-tiered algorithm had suspected EM for longer compared to those testing negative by all two-tiered algorithms (mean 7.4 vs 3.9 days; *P* = 0.002). Individual algorithm results and duration of suspected EM are shown in Table 7. When evaluating GSD MTTT and STTT positivity in these 92 cases, neither GSD MTTT nor GSD STTT was frequently positive within the first week, but by the second week, MTTT identified more cases than STTT, and after 2 weeks, both were frequently positive (Fig. 1).

**TABLE 7:** EM comparison between two-tiered algorithms^*a*^^,^^*b*^

Two-tiered algorithms	Tested positive			Tested negative			Significant difference?	
	N	EM at enrollment	Days with EM	N	EM at enrollment	Days with EM	EM at enrollment	Days with EM
		N (%)	Mean (median)		N (%)	Mean (median)	*P* value	*P* value
MC STTT	31	22 (71)	7.1 (6)	76	71 (93)	4.4 (4)	**	*
GSD STTT	24	19 (79)	7.7 (7)	83	74 (89)	4.3 (4)		**
GSD MTTT	38	30 (79)	7.4 (6)	69	63 (91)	3.9 (3.5)		**
Zeus MTTT	37	28 (76)	7.4 (6)	8	6 (75)	4.3 (3)		

^*a*^ A chi-squared test was used to test the proportion of participants enrolled with suspected EM, and a Mann-Whitney U test was used to test the days with suspected EM between those who tested positive and those who tested negative. Mean and median calculations exclude the cases that enrolled without suspected EM. Stars indicate the level of significance: *P < 0.05; **P < 0.01; ***P < 0.001.

^*b*^ MC, Mayo Clinic; GSD, Gold Standard Diagnostics; Zeus, Zeus Scientific; STTT, standard two-tiered testing; MTTT, modified two-tiered testing.

## DISCUSSION

This study confirms that two-tiered serologic testing algorithms using FDA-cleared assays are insensitive in early LD, particularly within the first 2 weeks of infection. Only 39% (42/107) of the first-draw samples collected from participants with early LD were STTT or MTTT positive. It also confirms that MTTT, regardless of test kit manufacturer, was more likely than STTT to detect early LD during the first 2 weeks of infection. This is consistent with several studies showing that MTTT is more sensitive than STTT among patients with EM (16). This study also confirms findings from an earlier study of the relationship between seropositivity and EM duration (17). In the current study, among participants enrolled with suspected EM, the likelihood of positive two-tiered testing results increased the longer they had the skin lesion prior to the first-draw serum sample collection; the duration of the EM lesion in those who were STTT or MTTT positive was ~3 days longer than those who were negative.

Participants with LD who reported constitutional symptoms, with or without concomitant EM, were more likely to test positive by STTT or MTTT than those without constitutional symptoms. Participants testing positive at the initial blood draw also had more symptoms and experienced them for longer durations (~5 days longer) compared to those testing negative. If the presence of constitutional symptoms reflects the immune system’s response to infection, then the increased probability of detecting a serologic response among participants with constitutional symptoms is not an unexpected finding.

Testing convalescent samples did not substantially improve the detection of LD. Paired acute- and convalescent-phase samples were available from 69 participants with early LD, all of whom were prescribed antibiotics for LD at the initial visit. Among these participants, GSD MTTT identified only two additional LD cases that were not MTTT positive at the initial draw, and seroconversion was not observed using either of the STTT algorithms evaluated in this study. Furthermore, individual serologic tests rarely demonstrated seroconversion. Seroconversion rates were 3% using the MC IgG immunoblot, 1% using the GSD IgG immunoblot, 3% using the GSD IgG ELISA, 3% using the GSD IgM ELISA, and 4% using the IgG+IgM ELISA. (Zeus second-tier assays were not run on convalescent samples.) This is consistent with our experience (15) and another study of 67 EM patients treated with antibiotics in which IgG seroconversion was only 4% (18).

### Implications for healthcare providers and clinical laboratories

There are limitations to serologic testing for evaluating patients with suspected early LD. Most notably, serology is poorly sensitive in early LD. Patients with a suspected EM lesion who lack constitutional symptoms and patients with a suspected EM lesion of short duration (<2 weeks) are unlikely to test positive using available STTT and MTTT algorithms; a negative test in these patients is especially unreliable. False negative results can lead to diagnostic and treatment delays that are associated with poorer patient outcomes (8). Seroconversion following antibiotic therapy is rare, and therefore, convalescent testing at 2–3 months after treatment is unlikely to provide clinically meaningful information.

Southern tick-associated rash illness (STARI), transmitted by *Amblyomma americana*, produces a rash similar in appearance to EM and can be difficult to distinguish from early LD clinically. Patients with STARI are expected to test negative using LD serologic assays. In regions with both Amblyomma and Ixodes tick populations, negative LD test results will not resolve the diagnostic dilemma.

There is also significant and potentially clinically meaningful discordance between the positive results produced by the two-tiered algorithms evaluated. Among the 33 first-draw samples collected from participants with LC Lyme disease that were positive by STTT, 67% were positive by both STTT algorithms, but MC STTT identified more early LD cases than GSD STTT. Among the 40 LC Lyme disease cases whose first draw samples were positive by MTTT, 85% were positive by both MTTT algorithms, while 93% were positive by GSD MTTT and 93% were positive by Zeus MTTT. Given that healthcare providers ordering laboratory tests have little control over which two-tiered algorithms are used by the testing laboratory, ordering providers should be encouraged to understand the performance characteristics and limitations of the particular two-tiered testing algorithms available to them. Providers should also be aware that negative test results do not rule out the diagnosis of early LD.

MTTT is more sensitive for the diagnosis of early LD compared with STTT. MTTT protocols also provide practical advantages for clinical testing laboratories. They are easier to administer and perform reproducibly because they are fully automated and eliminate the need to subjectively assess the presence/absence of an antibody-antigen band. Both steps can often be run in-house without needing to send samples to a reference laboratory, resulting in faster turnaround times and reduced costs (10). However, for healthcare providers, removing the immunoblot component sacrifices potentially useful information about the specific antigens against which the host immune response is directed. With this information, one can better judge the maturity and extent of the antibody response, which can be correlated with clinical features to improve diagnostic accuracy (16).

### Limitations

This study had several limitations. Participants were enrolled with signs and symptoms of early LD at clinical sites on Long Island and in Central Wisconsin, representing only two different U.S. geographic regions. Also, due to the variability in presentation of EM, it is possible that not all of the skin lesions suspected to be EM were actually EM (4). Similarly, constitutional symptoms attributed to early LD in this study are non-specific, and it’s likely that not all symptoms present at enrollment were due to early LD. Testing was performed at three different laboratories, which could have introduced variability to the results. Zeus second-tier testing was only performed on cases classified as LC Lyme and endemic controls, and (unlike the other assays) was not performed on the 62 cases that were not classified as laboratory confirmed by LDB. Finally, samples were not originally tested following the two-tiered algorithm protocols. Rather, all samples were tested using first- and second-tier tests, regardless of the first-tier test outcome, which is not standard practice. However, the two-tiered testing algorithm results were applied retrospectively to model standard practice.

### Conclusion

Collection of samples from well-characterized LDB participants with suspected early LD and endemic controls allowed for cross-comparison evaluations of the performance of various two-tiered serologic testing algorithms. Although MTTT confirmed more early LD cases than STTT, all STTT and MTTT algorithms evaluated were relatively insensitive in early disease. There was also substantial discordance between results obtained using different test kits on the same samples. Early LD patients with at least one constitutional symptom, with or without suspected EM, were more likely to test positive by STTT or MTTT. Among those with a suspected EM skin lesion, the likelihood of positive two-tiered test results increased with a longer duration of the lesion. For patients with suspected early LD, especially for those presenting without EM or without typical EM, there continues to be an urgent need for novel diagnostics that do not rely on serologic testing methods.

## Data Availability

The raw data supporting the conclusions of this article will be made available by the authors.

## AFTER EPUB

*[This article was published on 21 April 2026 with an omitted line in the Acknowledgments and incorrect footnotes in Tables 4 and 6. The Acknowledgments and Tables 4 and 6 were corrected in the current version, posted on 29 April 2026.]*

## SUPPLEMENTAL MATERIAL

Supplemental materials jcm.01187-25-s0001.docx Tables S1 and S2, and Additional test results.

ASM does not own the copyrights to Supplemental Material that may be linked to, or accessed through, an article. The authors have granted ASM a non-exclusive, world-wide license to publish the Supplemental Material files. Please contact the corresponding author directly for reuse.
