# Supplementary material for: Evaluation of standard and modified two-tiered testing algorithms using well-characterized early Lyme disease samples
Source: J Clin Microbiol. 2026 Apr 21;64(5):e01187-25. doi: 10.1128/jcm.01187-25 (PMC13170176; doi:10.1128/jcm.01187-25)
Supplement: Supplemental materials — Tables S1 and S2, and Additional test results. [file jcm.01187-25-s0001.docx]

| **FDA-Cleared Assays** | | **All Cases** | **Cases Enrolled w/ EM** | **Endemic Controls** |
| --- | --- | --- | --- | --- |
|  |  | N (%) | N (%) | N (%) |
| **First Draws** | | **N = 107** | **N = 93** | **N = 144** |
| **First-Tier ELISAs** | |  |  |  |
|  | C6 Peptide ELISA | 44 (41) | 34 (37) | 10 (7) |
|  | VlsE/pepC10 ELISA (Zeus) | 54 (50) | 41 (44) | 17 (12) |
|  | VlsE-OspC ELISA (GSD) | 40 (37) | 31 (33) | 0 (0) |
| **Second-Tier ELISAs** | |  |  |  |
|  | IgM ELISA (GSD) | 35 (33) | 26 (28) | 7 (5) |
|  | IgG ELISA (GSD) | 20 (19) | 17 (18) | 0 (0) |
|  | IgG+IgM ELISA (GSD) | 37 (35) | 29 (31) | 1 (1) |
|  | IgM ELISA (Zeus)* | 33 (73) | 25 (74) | 21 (15) |
|  | IgG ELISA (Zeus)* | 20 (44) | 14 (41) | 4 (3) |
| **Second-Tier Immunoblots** | |  |  |  |
|  | IgM blot (MC)** | 34 (32) | 25 (27) | 5 (4) |
|  | IgG blot (MC) | 8 (7) | 5 (5) | 1 (1) |
|  | IgM Blot (GSD) | 23 (21) | 18 (19) | 3 (2) |
|  | IgG Blot (GSD) | 7 (7) | 5 (5) | 1 (1) |
| **Second Draws** | | **N = 69** | **N = 59** | **NA** |
| **First-Tier ELISAs** | |  |  |  |
|  | C6 Peptide ELISA | 30 (43) | 24 (41) | NA |
|  | VlsE/pepC10 ELISA (Zeus) | 32 (46) | 23 (39) | NA |
|  | VlsE OspC ELISA (GSD) | 20 (29) | 15 (25) | NA |
| **Second-Tier ELISAs** | |  |  |  |
|  | IgM ELISA (GSD) | 16 (23) | 12 (20) | NA |
|  | IgG ELISA (GSD) | 10 (14) | 7 (12) | NA |
|  | IgG+IgM ELISA (GSD) | 20 (29) | 16 (27) | NA |
|  | IgM ELISA (Zeus) | NA | NA | NA |
|  | IgG ELISA (Zeus) | NA | NA | NA |
| **Second-Tier Immunoblots** | |  |  |  |
|  | IgM blot (MC) | 20 (29) | 16 (27) | NA |
|  | IgG blot (MC) | 7 (10) | 4 (7) | NA |
|  | IgM Blot (GSD) | 13 (19) | 10 (17) | NA |
|  | IgG Blot (GSD) | 4 (6) | 1 (2) | NA |

**Table S1**: Single-tier results for all 12 FDA-cleared assays. Number (%) of samples testing positive by each assay are shown. Only LC Lyme samples and Endemic Controls were tested with Zeus second-tier assays. For MC second-tier immunoblots, one IgM blot from a case and two IgM blots from controls were uninterpretable reducing the denominators.

*Denominators for cases are 45 and 34, respectively.

**Denominators are 106, 92, and 142, respectively.

Abbreviations: EM, erythema migrans; MC, Mayo Clinic; GSD, Gold Standard Diagnostics; Zeus, Zeus Scientific; ELISA, enzyme-linked immunosorbent assay; NA, not applicable.

| **Index** | **Enrollment Type** | **LDB Classification** | **MC STTT** | **GSD STTT** | **GSD MTTT** | **Zeus MTTT** | **MC STTT (M, G, M/G)** | **GSD STTT (M, G, M/G)** | **GSD MTTT (M, G, M/G)** | **ZEUS MTTT (M, G, M/G)** |
| --- | --- | --- | --- | --- | --- | --- | --- | --- | --- | --- |
| ***Samples testing positive with 4 two-tiered algorithms*** | | | | | | | | | | |
| 1 | With EM | LC Lyme (1) | **POS** | **POS** | **POS** | **POS** | **M/G** | **M** | **M/G** | **M/G** |
| 2 | With EM | LC Lyme (1) | **POS** | **POS** | **POS** | **POS** | **M** | **M/G** | **M/G** | **M** |
| 3 | With EM | LC Lyme (1) | **POS** | **POS** | **POS** | **POS** | **M/G** | **M** | **M/G** | **M/G** |
| 4 | With EM | LC Lyme (1) | **POS** | **POS** | **POS** | **POS** | **M/G** | **G** | **M/G** | **M/G** |
| 5 | With EM | LC Lyme (1) | **POS** | **POS** | **POS** | **POS** | **M** | **M** | **M/G** | **M/G** |
| 6 | With EM | LC Lyme (1) | **POS** | **POS** | **POS** | **POS** | **M** | **M** | **M** | **M** |
| 7 | W/O EM | LC Lyme (1) | **POS** | **POS** | **POS** | **POS** | **M** | **M** | **M** | **M/G** |
| 8 | With EM | LC Lyme (1) | **POS** | **POS** | **POS** | **POS** | **M** | **M** | **M/G** | **M/G** |
| 9 | W/O EM | LC Lyme (1) | **POS** | **POS** | **POS** | **POS** | **M/G** | **M/G** | **M/G** | **M** |
| 10 | W/O EM | LC Lyme (1) | **POS** | **POS** | **POS** | **POS** | **M** | **M** | **M** | **M/G** |
| 11 | With EM | LC Lyme (1) | **POS** | **POS** | **POS** | **POS** | **M** | **G** | **M/G** | **M** |
| 12 | With EM | LC Lyme (1) | **POS** | **POS** | **POS** | **POS** | **M** | **M** | **M/G** | **M/G** |
| 13 | With EM | LC Lyme (1) | **POS** | **POS** | **POS** | **POS** | **M** | **M** | **M/G** | **M** |
| 14 | With EM | LC Lyme (1) | **POS** | **POS** | **POS** | **POS** | **M** | **M** | **M** | **M** |
| 15 | With EM | LC Lyme (1) | **POS** | **POS** | **POS** | **POS** | **M** | **M** | **M/G** | **M/G** |
| 16 | With EM | LC Lyme (1) | **POS** | **POS** | **POS** | **POS** | **M** | **M** | **M** | **M** |
| 17 | With EM | LC Lyme (1) | **POS** | **POS** | **POS** | **POS** | **M** | **M** | **M** | **M** |
| 18 | With EM | LC Lyme (1) | **POS** | **POS** | **POS** | **POS** | **M/G** | **M/G** | **M/G** | **M/G** |
| 19 | With EM | LC Lyme (1) | **POS** | **POS** | **POS** | **POS** | **M** | **M** | **M** | **M** |
| 20 | With EM | LC Lyme (1) | **POS** | **POS** | **POS** | **POS** | **M/G** | **M/G** | **M/G** | **M/G** |
| 21 | W/O EM | LC Lyme (1) | **POS** | **POS** | **POS** | **POS** | **M/G** | **M/G** | **M/G** | **M/G** |
| 22 | W/O EM | LC Lyme (1) | **POS** | **POS** | **POS** | **POS** | **M** | **M** | **M/G** | **M/G** |
| ***Samples testing positive with 3 two-tiered algorithms*** | | | | | | | | | | |
| 23 | With EM | LC Lyme (1) | **POS** | NEG | **POS** | **POS** | **M** | NA | **G** | **M** |
| 24 | With EM | LC Lyme (1) | **POS** | NEG | **POS** | **POS** | **M** | NA | **G** | **M** |
| 25 | With EM | LC Lyme (1) | **POS** | NEG | **POS** | **POS** | **M** | NA | **G** | **M** |
| 26 | With EM | LC Lyme (1) | **POS** | NEG | **POS** | **POS** | **M** | NA | **M** | **M** |
| 27 | With EM | LC Lyme (1) | **POS** | NEG | **POS** | **POS** | **M** | NA | **M/G** | **M** |
| 28 | W/O EM | LC Lyme (1) | **POS** | NEG | **POS** | **POS** | **M** | NA | **M** | **M** |
| 29 | With EM | LC Lyme (3) | NEG | **POS** | **POS** | **POS** | NA | **M** | **M/G** | **M/G** |
| ***Samples testing positive with 2 two-tiered algorithms*** | | | | | | | | | | |
| 30 | W/O EM | LC Lyme (1) | **POS** | NEG | NEG | **POS** | **G** | NA | NA | **G** |
| 31 | W/O EM | LC Lyme (1) | **POS** | NEG | NEG | **POS** | **M** | NA | NA | **M** |
| 32 | With EM | LC Lyme (3) | NEG | **POS** | **POS** | NEG | NA | **M** | **G** | NEG |
| 33 | With EM | LC Lyme (3) | NEG | NEG | **POS** | **POS** | NA | NA | **M** | **G** |
| 34 | With EM | LC Lyme (3) | NEG | NEG | **POS** | **POS** | NA | NA | **M/G** | **G** |
| 35 | With EM | LC Lyme (3) | NEG | NEG | **POS** | **POS** | NA | NA | **M** | **M** |
| 36 | With EM | LC Lyme (3) | NEG | NEG | **POS** | **POS** | NA | NA | **M** | **M** |
| 37 | With EM | LC Lyme (3) | NEG | NEG | **POS** | **POS** | NA | NA | **M** | **G** |
| ***Samples testing positive with 1 two-tiered algorithms*** | | | | | | | | | | |
| 38 | W/O EM | LC Lyme (1) | **POS** | NEG | NEG | NEG | **M** | NA | NA | NA |
| 39 | W/O EM | LC Lyme (2) | NEG | NEG | **POS** | NEG | NA | NA | **M** | NA |
| 40 | With EM | LC Lyme (3) | NEG | NEG | **POS** | NEG | NA | NA | **M/G** | NA |
| 41 | W/O EM | LC Lyme (4) | NEG | NEG | NEG | **POS** | NA | NA | NA | **G** |
| ***Samples testing negative with all two-tiered algorithms*** | | | | | | | | | | |
| 42 | With EM | LC Lyme (5) | NEG | NEG | NEG | NEG | NA | NA | NA | NA |
| 43 | With EM | LC Lyme (4) | NEG | NEG | NEG | NEG | NA | NA | NA | NA |
| 44 | With EM | LC Lyme (3) | NEG | NEG | NEG | NEG | NA | NA | NA | NA |
| 45 | With EM | LC Lyme (3) | NEG | NEG | NEG | NEG | NA | NA | NA | NA |

**Table S2**: STTT and MTTT testing results of 45 LDB LC Lyme samples. Summary results of each two-tiered algorithm are shown, including whether IgM, IgG, or both IgM/IgG were positive on second-tier assays.

Abbreviations: MC, Mayo Clinic; GSD, Gold Standard Diagnostics; Zeus, Zeus Scientific; STTT, standard two-tiered testing, MTTT, modified two-tiered testing; EM, erythema migrans; LDB LC, Lyme Disease Biobank lab confirmed; positive, pos; negative, neg; NA, not applicable

LC Lyme categorizations: LC Lyme (1): Two-tier positive using VlsE/Pepc10 ELISA on first tier; LC Lyme (2): Two-tier positive using C6 peptide ELISA only on first tier; LC Lyme (3): 2 positive ELISAs (VlsE/Pepc10 ELISA and C6 peptide ELISA) and EM > 5cm; LC Lyme (4): IgG seroconversion on convalescent draw; LC Lyme (5): Blood PCR positive
